# The Patient Deficit Model Overturned: a qualitative study of patients' perceptions of invitation to participate in a randomized controlled trial comparing selective bladder preservation against surgery in muscle invasive bladder cancer (SPARE, CRUK/07/011)

**DOI:** 10.1186/1745-6215-13-228

**Published:** 2012-11-29

**Authors:** Clare Moynihan, Rebecca Lewis, Emma Hall, Emma Jones, Alison Birtle, Robert Huddart

**Affiliations:** 1 Institute of Cancer Research, London, UK; 2 Lancashire Teaching Hospitals NHS Trust, Preston, Lancashire, UK

**Keywords:** Patient interviews, Randomized controlled trials, Equipoise, Bladder cancer, Complex interventions, Qualitative methodology, Communication, Confusion

## Abstract

**Background:**

Evidence suggests that poor recruitment into clinical trials rests on a patient ‘deficit’ model – an inability to comprehend trial processes. Poor communication has also been cited as a possible barrier to recruitment. A qualitative patient interview study was included within the feasibility stage of a phase III non-inferiority Randomized Controlled Trial (RCT) (SPARE, CRUK/07/011) in muscle invasive bladder cancer. The aim was to illuminate problems in the context of randomization.

**Methods:**

The qualitative study used a ‘Framework Analysis’ that included ‘constant comparison’ in which semi-structured interviews are transcribed, analyzed, compared and contrasted both between and within transcripts. Three researchers coded and interpreted data.

**Results:**

Twenty-four patients agreed to enter the interview study; 10 decliners of randomization and 14 accepters, of whom 2 subsequently declined their allocated treatment.

The main theme applying to the majority of the sample was confusion and ambiguity. There was little indication that confusion directly impacted on decisions to enter the SPARE trial. However, confusion did appear to impact on ethical considerations surrounding ‘informed consent’, as well as cause a sense of alienation between patients and health personnel.

Sub-optimal communication in many guises accounted for the confusion, together with the logistical elements of a trial that involved treatment options delivered in a number of geographical locations.

**Conclusions:**

These data highlight the difficulty of providing balanced and clear trial information within the UK health system, despite best intentions. Involvement of multiple professionals can impact on communication processes with patients who are considering participation in RCTs. Our results led us to question the ‘deficit’ model of patient behavior. It is suggested that health professionals might consider facilitating a context in which patients feel fully included in the trial enterprise and potentially consider alternatives to randomization where complex interventions are being tested.

**Trial Registration:**

ISRCTN61126465

## Background

The Randomized Controlled Trial (RCT) has long represented the gold standard for clinical scientific investigation, requiring objectivity, validity and fully informed consent. Phase III trials normally require large numbers of participants to detect clinically meaningful differences [1,2]. Complex RCTs can present difficulties, to the extent that low levels of recruitment can cause individual trials to close prematurely [3-5].

Few studies examine how to compare radical surgery to a relatively non-invasive treatment, such as radical radiotherapy [6]. The nature of the organizational context and everyday working practices of trial personnel that may influence precision, validity and accrual rates of RCTs are seldom focused on [7].

Studies suggesting solutions to poor recruitment in cancer trials usually do so from the service provider’s view, premised on a ‘deficit model’ [8]. Patients become ‘the problem’ [8], ‘misunderstanding’ concepts such as equipoise and randomization [9-11], risks and benefits of participation [12,13], trial options and procedures in general [14,15], resulting in a potential violation of the Declaration of Helsinki [16,17].

Researchers have embedded qualitative studies within trials to investigate participants’ perspectives [11,18-20]. These and other studies have advised better recruitment to RCTs by addressing communication [5,15,16,21-23], physicians’ reluctance to randomize [5,15,23-26], an inability to describe equipoise [5,11,20], time restraints [12], physicians’ misconceptions [12], patients’ [5,27] and physicians’ [5,23] treatment preferences and patients’ trust in their physicians [5,19,20,28-30]. The patient deficit model generally persists, however.

This paper focuses on a randomized trial of Selective bladder Preservation Against Radical Excision (cystectomy) in muscle invasive bladder cancer (SPARE) and the ways in which perceived trial procedures and communicative processes may have affected ‘informed consent’ and forestalled patient participation.

### The context of the SPARE trial

SPARE (CRUK/07/011) was a phase III non-inferiority RCT in muscle invasive T2/T3 transitional cell carcinoma of the bladder, with an initial feasibility stage to assess recruitment rates and compliance with assigned treatment. Embedded within the feasibility stage was a qualitative patient interview study. The trial was designed in consultation with patient representatives and approved by the South-East Multicentre Research Ethics Committee.

Target accrual for the two-year feasibility stage was 110 patients. The main phase III trial would aim to recruit up to 1,015 patients, with an endpoint of overall survival. Patients aged 18 and over with histologically confirmed T2/T3 N0 M0 after transitional cell carcinoma of the bladder receiving neo-adjuvant gemcitabine-cisplatin chemotherapy, with satisfactory hematological profile and liver function tests and who were fit for both trial treatments were eligible.

**Figure 1:**
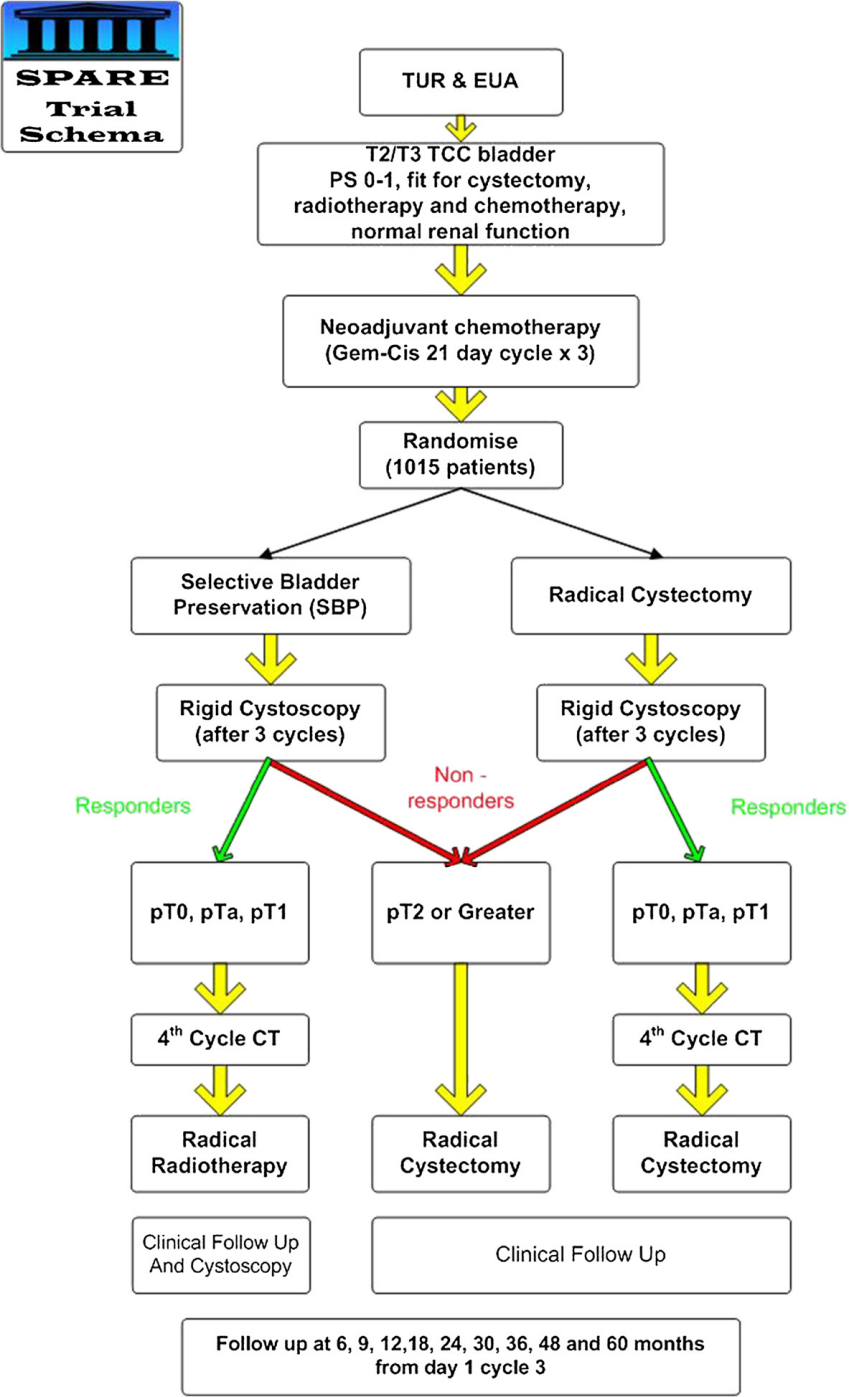
**SPARE Trial Schema.** TUR – Transurethral resection, EUA – Examination under anaesthetic, TCC – Transitional cell carcinoma, PS – Performance status, Gem-cis – Gemcitabine – cisplatin chemotherapy, CT – chemotherapy.

Patients were centrally randomized between radical surgery (cystectomy) following neo-adjuvant chemotherapy, and selective bladder preservation (SBP) where definitive treatment (radiotherapy or cystectomy) was decided based on response to neo-adjuvant chemotherapy (Figure 1). Consenting patients were randomized between Day 1, Cycle 2 of chemotherapy and Day 1, Cycle 3.

Participants completed three three-week cycles of neo-adjuvant chemotherapy, followed by cystoscopy. If the tumor was down-staged to less than pT2, the patient then received a fourth cycle of chemotherapy and proceeded to allocated treatment, that is cystectomy or, if randomized to SBP, radiotherapy. Without down-staging, patients in both groups received cystectomy without a fourth cycle of chemotherapy.

### Patient pathway and identification

**Figure 2:**
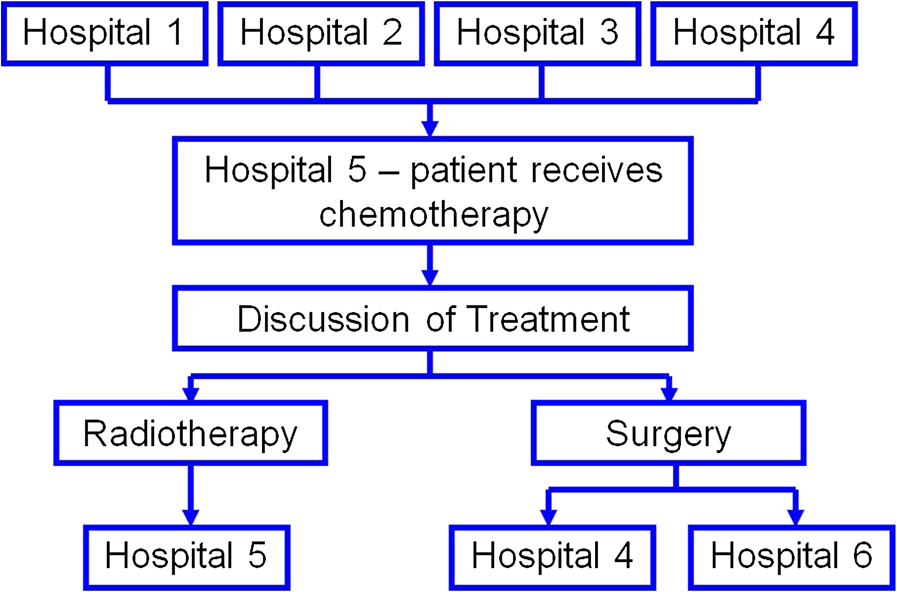
Patient diagnosis and treatment referral pathway.

Patients were referred by General Practitioners (GPs) to urologists who diagnosed bladder cancer and subsequently referred them to clinical oncologists. Due to the complexity of cancer treatment networks in the UK, some patients traveled from the diagnosing hospital to another center for definitive treatment (Figure 2).

Center staff explained the trial and provided information sheets that had been carefully scrutinized for patient acceptability. Patients were given time to consider participation. Consent was obtained prior to randomization. The qualitative study was designed to illuminate problems and reasons why patients accepted or declined randomization.

## Methods

### The Qualitative Study

Prior to starting definitive treatment, accepters and decliners to randomization were approached and provided with a qualitative study information sheet, as well as receiving an explanation regarding the rationale of the study. Consent was obtained to pass names and contact details to the researcher (CM) who contacted patients to ascertain interest in being interviewed in a venue of their choice, soon after the invitation to enter the study. Patients who agreed to participate gave consent and were interviewed consecutively until no new themes were forthcoming.

Patients were asked: ‘Please tell me about your bladder cancer experience’ and their perception of trial procedures was explored, including:

Randomization and equipoise

Medical terms

Medical procedures

Information given and received

Communication

Decision making

Reasons for accepting or declining

A social scientist (CM), the SPARE trial manager, (RL) and the SPARE Quality of Life Coordinator, a psychologist by training (EJ), ‘rated’ and interpreted the patients’ accounts. CM conducted all the interviews to ensure standardization. The paper was circulated to all SPARE authors for comment prior to submission.

Study findings were discussed during regular Trial Management Group meetings; were disseminated by letter to trialists and by presentation to SPARE investigators in an effort to improve informed consent levels and recruitment rates, but no formal assessment was made of how that information impacted on recruitment procedures.

### Method of analysis

Recorded interviews were semi-structured, in line with a ‘Framework Analysis’ [31] that allows for the inclusion of respondents’ own concerns as well as answering specific topics. The choice of topics was informed by clinical concerns and previous research in this area of investigation. Topics were covered in a flexible manner; patients were probed if they did not arise spontaneously in the interview. Analysis was an iterative process, allowing for the follow-up of emergent findings. Items were systematically coded, initially depending on their face value, followed by conceptual coding leading to over-arching themes and deviant accounts. The method of constant comparison was used, where interpretations are made by checking patients’ perceptions within and across verbatim transcripts [32]. Interpretation relied on patient context, literature and field notes. The researchers met regularly to compare coding and interpretation to ensure reliability. Disagreements (such as they were) were resolved and at times, points of discussion were taken to relevant experts to verify findings. In these cases, the coding framework was refined and where applicable, reapplied to the data.

## Results

SPARE opened in June 2007. In February 2010, with 45 patients recruited, it closed in view of the unfeasibility of the planned phase III trial due to insufficient eligible patients [33]. Patient interviews took place between June 2007 and January 2009, by which time 29 patients (of 114 eligible) had been recruited into SPARE from 12 centers.

**Figure 3:**
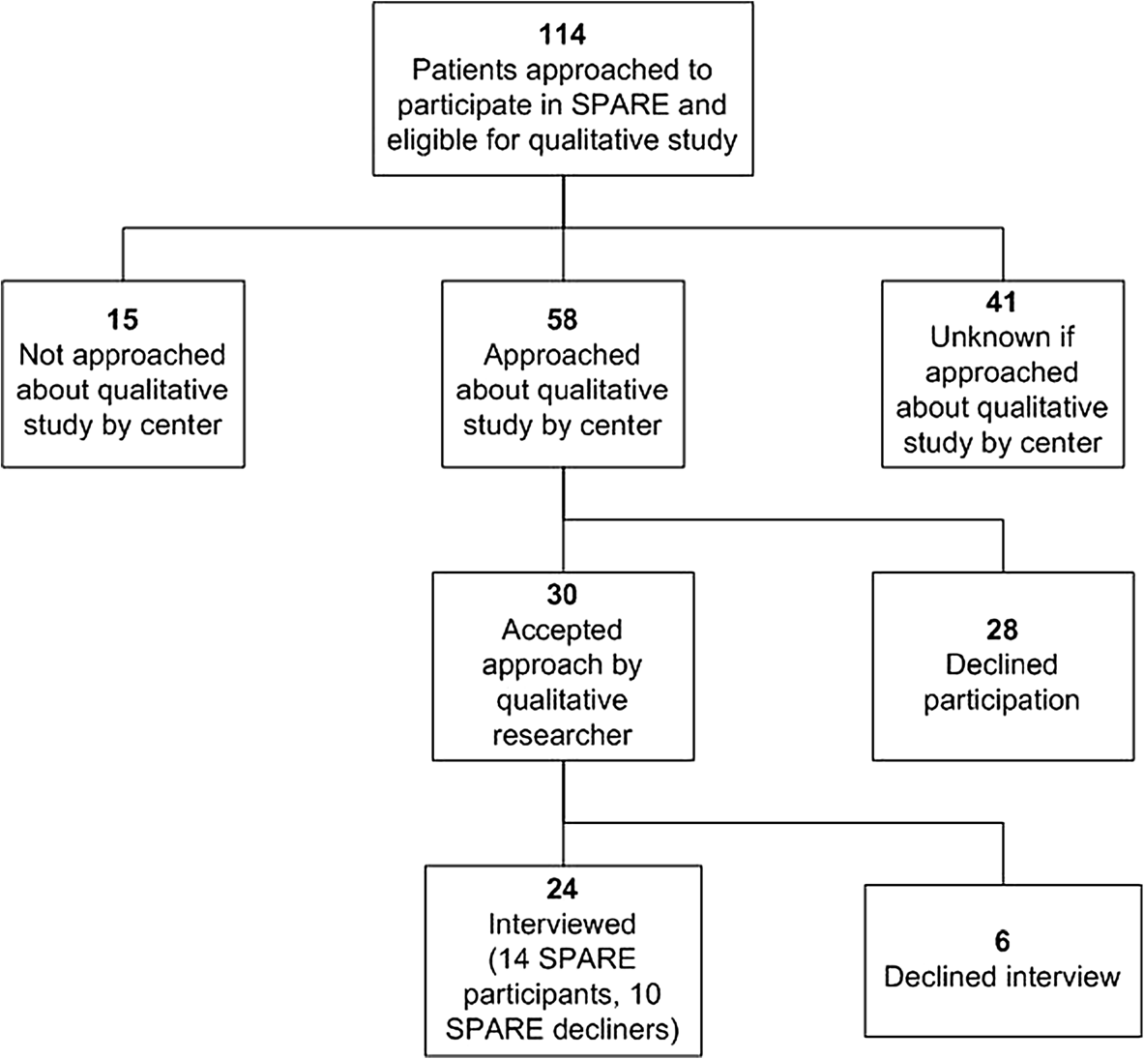
Qualitative study participant flowchart.

A total of 24 patients (21%) were interviewed (Table 1) – 14 accepters (8 cystectomy, 6 SBP), 2 of whom went on to decline their randomized treatment (accepter/decliners – both allocated cystectomy) and 10 decliners, including 1 patient and his partner, whose comments were recorded and analyzed together with her husband’s (Figure 3).

**Table 1:** Participant characteristics

	**Accepters (n = 12)**	**Decliners (n = 10)**	**Accepters/Decliners (n = 2)**
Mean age (yrs)	65 (SD 5.6)	71 (SD 5.7)	74 (SD 2.8)
No. males	11 (92%)	7 (70%)	1
Married/Cohabiting	11 (92%)	9 (90%)	1
Upper socio-economic status	9 (75%)	9 (90%)	0

Socioeconomic status is defined according to Office of National Statistics (UK) categories: 1, 2, 3 NM = non-manual, skilled jobs (Upper Socio Economic Status); 3, 4, 5 M = manual, unskilled jobs. Retired and unemployed are categorized as “previous employment” or, when “no job,” are categorized by employment of head of household.

Of participants who had consented to SPARE, five were interviewed prior to definitive treatment (median of 13 days before (interquartile range (IQR) -36 to −8)), eight were interviewed following treatment (median of 51 days afterwards (IQR 31 to 57)). One patient was interviewed during radiotherapy treatment. Ethics approval was not sought to collect treatment details for patients refusing SPARE; however, the timing of the interview relative to treatment did not appear to differ for decliners. The majority were interviewed at home, across the United Kingdom. Patients’ characteristics are seen in Table 1. Patients were asked: ‘Please tell me about your bladder cancer experience’ and probed to cover the relevant topics while welcoming patients’ own comments.

One overarching theme emerged, namely: ‘Confusion, ambiguity and alienation: professional and structural difficulties in communicating to participants’. Sub-themes included ‘Communicating trial concepts and procedures’, ‘Inter-relational communication’ and ‘Administration as communication’.

Patients were satisfied with some aspects of their experience and simultaneously dissatisfied with others. Satisfaction appeared to lie in respect, trust and connectedness with health personnel and the medical enterprise.

‘.he/she’s a (doctor) who instills confidence in you .….and that is one of the things that was a consideration when push came to shove and I had to decide which way it (participation) was going….”

— Mr Brown - Accepter

“the nurses and that are wonderful and the fact that you can ring them anytime, you have a number that you can ring day or night just really to talk to someone….tell them if there is anything that you are worried about and they will say.’come down’ or whatever”

— Mrs Sandy - Decliner

All but four participants experienced some aspect of confusion, including problems with the UK National Health Service (NHS) structure and general administration, the design and procedures of the trial, concepts of randomization, equipoise and personal communication. Although there was an indication that decliners to participation were in higher social class categories (Table 1), there was no consistent association between patients’ participation, perceived confusion and patient characteristics.

### Communicating trial concepts and procedures

Patients displayed what may be perceived as ‘poor understanding’ of trial procedures and concepts. This did not appear to rely on cognition, however. Patients’ accounts suggested that information giving was often sub-optimal [8] and/or understanding unverified, highlighting the crucial role that communication plays in obtaining ‘informed consent’ [5,11,18-21,29,33-36].

There was little that revealed participants’ interpretation of equipoise although there was evidence that patients and partners who articulated their understanding of the concept had access to the medical enterprise.

“Seeing all the specialists helped me more than I can say…my wife (a medical practitioner) and I asked to see anyone who might treat me ……and Dr X indicated that not enough was known about the two separate treatments (surgery and radiotherapy)…and if the chemo didn’t work I would then go ahead with the surgery.”

— Mr Van - Accepter

An explanation of equipoise was usually perceived to be absent in the information process, calling for an essential need to validate participants’ understanding prior to obtaining consent.

“They told me they were doing the trial for mortality…to see who lived longer….but nobody used the word ‘equipoise’ or that they literally don’t know which is better…”

— Mr Brown - Accepter

The need to believe in expert physicians and an inability to accept medical uncertainty is documented [20,36]. Moreover, studies have documented the ways in which patient preferences over-ride equipoise and randomization [5,13,30,36]. Even when trial concepts are explained (and perceived correctly), patients may reject advice for personal but rational reasons [37]. Below, Mr Baker voices his incredulity as he faces the ramifications of uncertainty while striving to maintain control over his own decision-making in the name of quality of life.

“He/she told me about the trial… It was something that (the doctors) couldn’t say what the best (treatment) was…I understood the meaning of balance…but I am an active, fit 63 year old man who travels…when I have anything to do with the medical profession they have said ‘I think …this is the treatment’ .( ) it was the first time that it became obvious that somebody wasn’t going to say that this is the best treatment for you ( ), you are left to make up your own mind…that is how I ended up going for the radiotherapy ( ) but it was still a preference rather than knowing it was right ( ) going into the SPARE trial, this option would be taken away from you.”

— Mr Baker - Decliner

Below, Mr Plane portrays the uncertainty inherent in any randomized trial, which often undermines people’s belief in medical progress. It is for this reason that randomization was often refused in a context where surgery was offered against a less radical option.

“…it (equipoise) was never explained to me …it’s the first that I have ever heard it…and if it had it would not have changed my mind … I will tell you why because they were doing that sort of surgery one hundred years ago…if they don’t like it chop it out …we have gone a bit further than that these days and I personally would explore lots of other routes.”

— Mr Plane - Decliner

Physicians find the concept of equipoise difficult, both because of personal preference [24,25], and the difficulties of explaining the uncertainty prevailing in any form of randomization, [25,26]. While differing options were appreciated, they might also cause confusion.

“I was blinkered by specialists who thought theirs was the one to follow!…The surgeon said ‘let’s operate’, he/she was quite bullish…’no trial, no options’, ( )…and this was before the SPARE trial was explained to me…I naturally thought (operating) was my only option…it was confusing.”

— Mr White - Accepter

Patients’ decision-making is known to be affected by the initial physician encounter where a special interest shown by ‘experts’ is perceived, appreciated and advice duly accepted [13,30,36]. While many of the participants in this study reiterated this, early advice was often discarded for reasons such as altruism.

“He/she (surgeon) felt that surgery was the most successful way of combating (the disease) …he/she said the quality of life is much more important (then I was told about SPARE)…I was relying on his/her approval and blessing (to participate)…( )…courtesy alone apart from his/her expertise…because he/she very kindly had brought me through from my initial treatment to this stage… I very much appreciated (it) ( )…but I wanted to help medical science.”

— Mr Marks - Accepter

Busy clinics, run with insufficient resources, are shown to underlie suboptimal communication and trial consent [16,38]. There was no dedicated, trial-specific funding to support recruitment to SPARE (other than access to National Institute for Health Research (NIHR) funded National Cancer Research Network resources). Moreover, the very structure of SPARE where patients moved from one treatment to another, may have accounted for a break in continuity and trial communication in general. Be that as it may, clinicians’ preferences and the impact of one careless comment made by clinicians not involved in the trial could jeopardize the consent process, especially when a patient was mindful of quality versus quantity of life.

“I was convinced that I was going to have my bladder out because that’s what Mr/Mrs X told me …I hadn’t been told about the trial …then I was told that I could have radiotherapy by a junior doctor…I was confused, it (a change of mind) wasn’t what I expected of doctors! … I saw the consultant who told me about SPARE …by this time my head was full of this young man/woman telling me that I might not necessarily need surgery…( ) and I am all for that and I would go for it if it only meant another ( ) 5 or 10 years rather than another 20 years because you are normal, aren’t you?”

— Mrs Sandy – Decliner

There were little data in this and other studies suggesting that randomization is perceived as an ethical follow on from equipoise or that bias is avoided through random allocation [36]. ‘Misunderstandings’ were sometimes perceived, however, to relate to a paucity of information, too much information or a simple misconstruing of concepts [19-21]. Often, as in other studies, randomization was perceived haphazardly as patients strove to make sense of their involvement in the trial process while questioning scientific principles [18,19].

“No, I haven’t got a clue (what randomization means) …it’s lack of information…(… ) they may at random…oh well ‘we’ll give him a needle in his arse…then we will stick one in his leg’ and just stick it in at random… without reason.”

— Mr Meadow - Decliner

Patients’ preferences and idiosyncratic beliefs about trial processes had little to do with the reality of SPARE. Below Mr. Brown, a football supporter and research advocate, knew that while the comparison between treatments underpins randomization, his reasons for participation lay in joining ‘the team’ or not, in his quest to ‘up the numbers’ - not to be allocated treatment. Like others, he intended to withdraw if his preferred treatment was not forthcoming.

“Randomization?… names out of a hat…it’s like a draw for the football…you draw out a team, the home team…I am the team that is playing with (SPARE) and that’s the randomization…preferably (computerized)…. they press a button and the computer would rattle around and come up with a name in bright lights with stars around it …( )by joining the group I have added to the numbers for research purposes ( ) but if I had been randomised to radiotherapy I would have dropped out…(…) I didn’t tell anybody.”

— Mr Brown – Accepter

Bias created through withdrawal post-consent is not reported in trial literature. However, the impact of withdrawal on a non-inferiority trial, such as SPARE, can have a deleterious effect when analyzed on an intention-to-treat basis [39]. Mr Brown goes on to question the veracity and validity of SPARE having scrutinized the information sheet. There was no evidence that his perceptions had been challenged.

“ (…) Three out of the four (options) that are available ended the same way with surgery…so I don’t see what randomization has to do with it …I didn’t see after reading the documentation on SPARE… what leaving or staying; what difference it would make…( )randomization wasn’t going to make any difference to my treatment.”

— Mr Brown – Accepter

The rare belief that withdrawal was impossible indicated that either information sheets had not been appreciated or trialists had not clarified patients’ rights [16].

“(Once randomized) you couldn’t volunteer for the other (treatment) because you only got that if you went into the trial and got away with it through randomization.”

— Mr Hall – Accepter

One of the endpoints of the feasibility study was adherence to treatment. If it had not been explained that patients may receive surgery in the SBP group, adherence to treatment could be compromised. Several patients had seemingly not understood that randomization included the option of surgery. Surgery was sometimes rightly perceived as the standard treatment, and randomization as a likelihood of having radiotherapy, not as a valid method of comparing treatments.

“He said ‘you will be selected to continue within the trial at random…there is a chance that the computer will say ‘you are not going any further in the trial’, at which stage you will have surgery to have your bladder removed. (…)…To my limited knowledge it would be chemotherapy and radiotherapy…(…) as treatment for cancer (…) I would be picked from the computer to continue on the trial to have chemotherapy and radiotherapy or leave ( ) and have surgery.”

— Mr Clarke – Accepter

While patients ‘misunderstood’ or queried trial design and procedures, their preferred treatment was often perceived as an inevitable outcome. The notion that treatment is individually ‘chosen’ through randomization is reported elsewhere [10,12,13,18,20]. While patients’ rights may not have been adhered to, nor patients’ beliefs regarding the trial procedures checked or challenged, the quote below highlights a belief that individual wishes are realized through the trial process. Indeed, cynicism was often palpable as doctors were perceived to collude with patients’ best interests while serving their own.

“I think there is an element of accommodation in ‘randomization’…(I got radiotherapy and I wanted it!)…they are doing it for their benefit as well as your benefit and they want their results to be proven.”

— Mr Hall – Accepter

Patients’ understanding of SPARE and the procedures inherent in randomized trials often appeared to be faulty but evidence of verification of trial knowledge was conspicuously absent. The obvious need for vigilance by trialists was compounded by the ways patients recounted a separation between themselves and all that the SPARE trial encompassed.

### Inter-relational communication

The patients’ sense of alienation was evident. Feelings of isolation, loss of control and powerlessness underwrote involvement in the trial process. Patients’ utterances were laced with words such as “feeling alone”, “no control”, “being a guinea pig” and “having no say”. Studies corroborate the importance of ‘a relationship’ between health professionals and patients and the ways in which ‘bad communication’ may undermine trust [19,21,40-42] and prevent participation in trials [40]. Research has, however, placed ‘good communication’ in the arena of information giving where full disclosure is advocated [43]. In this study, as in others, devices such as information leaflets or hurried verbal information often created rifts between patients and physicians [36]. Below is a quote from a man who was ‘in charge’ of his life but nevertheless sought connection - ‘a conversation’ with his doctor.

“He/she (the surgeon) says ‘well life expectancy is three to five years’ I thought that was out of order…boom boom… it all came out …It’s a minefield…( )…whether he/she (Mr/Mrs X) could have done that (given diagnosis and prognosis) in a more caring way…or just slung me out in the corridor I don’t know (…) giving me all those leaflets was a bit of a shock (…)…I wanted a conversation with my doctor.”

— Mr White – Accepter

While medical progress was anticipated, a significant number of patients felt undermined, resulting in mistrust in the medical enterprise. This was articulated by patients across the sample, when they failed to understand the ‘language’ of trial procedures.

“Cystoscopy”… The name for having your bladder out? …Was it “endoscopy’ or?…Yeah…I always worry when people start throwing long words at me …they are attempting to confuse me…I want to know it in lay terms like ‘I am having surgery’… like ‘the bladder out’…the paperwork always related it to medical terms.”

— Mr Brown – Accepter

Alienation was attributable in part to research overload [36], information overload [14], a perceived lack of information [14] and a sense of coercion [15] undermining autonomy that, in some cases, affected decision making. Patients in this study were sometimes asked to enter up to four studies. Difficulties regarding assimilation of information and an obvious disconnection between trialists and their patients were recounted, to the extent that patients misunderstood their position in the consent process [14,16,36].

“When I saw ( ) the nurse he/she said about me not having the treatment and I said well I haven’t refused it…and I didn’t know enough about it . . .all I knew is ( ) you are going as a human guinea pig and they can do what they like…I said “no I didn’t refuse it’…(and) they said ‘yes’…but now I said ‘you have asked me if I am having chemo and now you have told me that I can’t go in for it anyway so why are you wasting my time’?…( )…and I said ‘that (was) a bit of a waste of time then wasn’t it?…I don’t know if he/she was talking about another treatment…I was asked to do umpteen things and quite honestly I didn’t understand why or what she was on about…when I said that they said ‘well it’s all written down there in the info sheet’ and I said ‘yeah, but it’s not very informative.’ ”

— Mr Meadow – Decliner

Mr Meadow’s ‘muddle’ regarding SPARE and his consent into the trial was encased in a sense of confused powerlessness, distrust and the ways that information received can be perceived as too little, but could also be construed as too much. In fact, he had apparently refused SPARE. Just as he articulated a loss of control despite his para-medical experience, the ability to hold down a serious job, and the experience of previous illness, others reiterated a sense of powerlessness in various ways. Thus, while withdrawal was often influenced by individual treatment preferences, a sense of control could be gained by reneging on consent.

“(…) but the only thing I don’t understand is when you are pulled out of the computer…that’s when the problem started (I withdrew) ’you will be picked out at random’ was what they said…and I had no more control…I got out.”

— Mrs Wall – Accepter/Decliner

Sometimes a sense of loss of control and perceived coercion led to feelings of isolation and disrespect for trialists. In such cases, patient autonomy and ethical practice were not upheld, [15,16], often due to a perceived lack of time to consider participation [5,12,13,16,20] during ‘consent discussions’ [12]. Below, Mrs Wall speaks of the way she perceived herself as sidelined in the clinician’s hurried quest to obtain consent, but with ineffectual consequences.

“(…) I thought well (the doctor) was pushing me (to consent) when he/she shouldn’t have been…and that really annoyed me…and I saw him/her twice and each time I thought those forms are far more important than how I was…he/she is quite abrupt…( ) I suppose the doctor was really talking to my son more than me ( ) I felt isolated.”

— Mrs Wall – Accepter/Decliner

Perceived ‘coercion’, such as illustrated in the quote above, was experienced and articulated in various ways. Below Mr Woods recounts the ways in which a physician who, no doubt in good faith, intervened in the wrong context and at the wrong time, portraying a schism between himself and his professional colleague, while creating confusion, anxiety and the patient’s need to establish autonomy.

“On Saturday night, (at home) Dr. Y rung me ( ) the way he/she spoke to me on the phone I just didn’t need it…I was alright as I was …he/she really give me a bit of an ear bashing…’ Mr X shouldn’t have told you this’… I said, ‘ I am only telling you what he told me’ …. I have never seen that doctor again…. He/she said ’you have got to do this…you have got to do that’ and I said ‘well I haven’t got to do anything…if I don’t want to go through with something’ I said…’I won’t go through with it’…(… )… it really upset me a bit ….”

— Mr Woods – Accepter

When researchers fail to deliver cohesive medical messages, patients become anxious. The demeanor of staff could be heard to create intra-familial problems and ‘bad’ feelings, including guilt and neglect as a result of an uneasy alienating atmosphere.

“I had a dreadful argument with Peter (son)…(…)…I said ‘I have had a phone call from the hospital and your father is going to have chemotherapy, radiotherapy’…’no he isn’t…I have been told differently’ (he said)… (…) ‘he is going to have the bladder out’…(…)…Confusing…I had been speaking to one Susan and Peter had been speaking to another Susan ( )…. Frank had been picked (randomized) to have his bladder out and he had said he didn’t want the bladder out, (because it was disease-free) he wanted radiotherapy ( ) we could feel an atmosphere…the doctor and the nurse didn’t like it…(…)…he/she didn’t want to speak to me and Frank felt guilty.”

— Mrs Hanson, wife of Mr Hanson - Accepter/Decliner

Difficult situations were compensated by ‘good’ accounts concerning staff, corroborating the necessity to engage in a personal connection with patients if only to enable a sense of inclusion in the hospital process.

“If I am going to give anybody any special praise… … the receptionist at the chemo clinic…he/she was pleasant, she would laugh and have a joke. He/she was well organised,…(…) absolutely superb…it makes the biggest difference …he/she made me welcome.”

— Mr Brown – Accepter

Favorable perceptions of personnel are pivotal to creating and maintaining trust and facilitating connectedness and mutual respect [35,36,41,42] leading to a sense of cohesion and autonomy. Previous work has focused on the ways in which hospital personnel give emotional care that is ‘hidden’ and seldom given credence [44]. Just as health workers are involved in unspoken emotional ‘work’, patients too may be immersed in ‘working the system’.

### Administration as communication

Although not the focus of this research, patients spontaneously indicated the need to ‘work’ their way around NHS waiting times and hospital administration. There is little in the literature that indicates the impact of this on patients’ trial behavior. Patients in this study often criticized their need to ‘work’ against ‘bad administration’, sometimes affecting trial decisions. This did not appear to rely on patient characteristics.

The majority of patients in this study responded quickly to symptoms. While there is evidence that this sense of urgency is mirrored by GPs and tertiary hospitals [45], treatment delay among this sample engendered anxiety, anger and scepticism while UK DOH guidelines [46] were acknowledged and criticized. Mr Hall recounts the way in which long waits were followed by the availability of instant treatment in the SPARE trial, determining his decision to consent.

“the whole handling (…) was crap…um the issue of getting to the hospital, getting the cystoscopy done…that was the maximum time… I went to (the doctor) in June and we didn’t do the camera until September ( ) we don’t start the treatment until December so its six months…( ) when I have got something wrong with me because I am bleeding (and I’ve been told that my tumor is aggressive)…to wait for any action to take place is not right …I still feel angry…they are playing lotto with your life…it’s all down to the targets and the way things are structured …(…) then I met Dr Y (who told me about SPARE) and I said ‘when can you start? ’We can start the chemo on Monday’. ‘OK , that was my main reason for going for the trial.”

— Mr Hall – Accepter

While patients were loath to criticize, difficulty at the hands of administrators was evident. Below, Mr Plane explains the ‘muddle’ he faced, and the potential to jeopardize trust and respect, if not participation.

“we would like you (to come) next Tuesday 15^th^ and Tuesday is the 14^th^…and so you would have to ring afterwards and say ‘do you want to see me on the Monday or the Tuesday’…and the other thing, I was half way through my chemo cycle and I ( ) got a call from Elsewhere hospital…‘can you make it down here at 1 o’clock next Tuesday?’ and I said ‘yes why’?…he/she said ‘because doctor what’s-his-name is down here, and we do your cystoscopy’…so I thought ‘oh well right’…in between that and going I had a word with my …oncologist and he/she went up the wall. ‘Why (…) half way through your treatment?…There is no point’…he had told me the 15th March, not the 15th of February…I would say to others ‘you have got to keep a weather eye open on what’s going on’ … I got three letters … two posted on the same day, one the day after giving me three different appointments to see the oncologist …( )…it might make others anxious!”

— Mr Plane – Decliner

The complicated logistical problems inherent in the patient pathway of SPARE (Figure 2) meant that some patients spoke with multiple physicians and traveled from the diagnosing hospital to another center for definitive treatment [47]. This jeopardized communication and could cause treatment decisions to be arrived at covertly.

“My treatment extends over four different counties…four hospitals…dealing with me )……it’s difficult to know what hospital is doing what…if all the procedures could have been carried out in one area…I would see the anesthetist who was not going to be my anesthetist ( ) …I had one doctor’s name on the admissions letter…one surgeon’s name on (it)…I was interviewed by another surgeon, on my discharge, there was a third doctor’s name and then a fourth doctor’s name ( ) and I wouldn’t have chosen radiotherapy because the nearest radiotherapy is (elsewhere) which is about an hour and half’s drive for a 10-minute treatment…’

— Mr Brown – Accepter

Mr Brown, like others, seemed unable to discuss his misgivings with clinicians, but demonstrated the way in which patients wish to contribute to science, despite it being perceived as a context filled with ambiguity and confusion.

## Discussion

This study highlights the perceptions of patients invited to enter a complex intervention trial that was curtailed because of low recruitment. While it was not always possible to make obvious links between trial procedures and patient participation, qualitative data have shown how sub-optimal communication in all its guises leads to confusing messages, alienation of patients and groundless decision-making, potentially compromising trial validity and ethical conduct.

Despite every effort to conduct a high quality trial, there was an inherent difficulty in managing a ‘high stake’ randomized trial such as SPARE where surgery was compared with radical radiotherapy. Such a study did not lend itself to explaining ‘equipoise’ and ‘random allocation’, the former being particularly difficult for clinicians to explain and patients to accept. Physicians’ preferences and the difficulties of ‘equipoise’ have been shown to undermine confidence and optimal recruitment [5,11,24,36,47], and challenge the traditional roles of doctor and patient [23]. Furthermore, an integration of what have been perceived as contrasting and competing roles of researcher and physician is required [23].

This study suggests that a debate as to whether RCTs are appropriate in the evaluation of complex interventions may be fruitful. Alternative strategies that take account of physician and patient preferences may help to provide a means of assessing the efficacy of complex treatments.

An aim of SPARE was to assess recruitment rates in a multi-center context. This took place in the absence of dedicated trial specific resources at the centers involved. Thus, as with most non-commercially funded RCTs in the UK, there was little leverage with which to influence the trial behavior of the many health professionals working with SPARE, although problems and suggested solutions were disseminated regularly. Our data reflected problems reported in previous studies. This raises questions about how research into trial recruitment is disseminated within modern health services and how trialists can be informed of and deliver best practice [5,12].

Structural issues and logistical problems inherent in the NHS are likely to have impacted on patient and physician trial behavior. The complicated treatment pathway in the SPARE trial (Figure 2) seems to have prohibited personnel from giving comprehensive explanations and consistent support regarding concepts and trial procedures. This helps to explain patients’ observed confusion and potentially uninformed consent, not withstanding that SPARE had to involve non-trial staff in the recruitment process who may have been unwilling [48] and/or less skilled at approaching patients and explaining trial concepts, such as equipoise.

Our findings are replicated in a further SPARE qualitative study that was conducted in real time [47]. Paramasivan *et al.* revealed that trialists had difficulty in articulating the complex trial design, leading to a palpable breakdown in communication. Information was given in complicated and erroneous terms, reflecting physicians’ preferences. We propose that attention be focused on training trialists who are involved in recruitment to complicated trials, both in terms of communication processes and on the assimilation of complex trial pathways experienced as a consequence of the current structures of the NHS. The ProtecT study [11] has demonstrated how this can be achieved in a similarly complex RCT involving surgery.

Participants in SPARE seemed to lack comprehension of trial procedures but this was not premised on a deficit model [13]. Study participants indicated that their understanding of randomization and equipoise were not necessarily verified. Both concepts are highly complex and require thought about how their meaning is communicated [37,40]. However, simple provision of clear factual information is often not sufficient to ensure ‘informed consent’ [5,13,30,36]. Patients in this study and others are known to interpret particular terms differently from what is actually said by clinicians [49,50], highlighting the importance of verification to achieve valid informed consent. As our data show, even if patients understand the scientific perspective, they may not accept its validity or the offer to participate in trials. They can rely instead on other factors, such as their own preferences associated with their everyday lives, both perspectives being rational and reasonable in their own terms. [38,49,50]. Research that investigates individual encounters between patients and their doctors remains essential.

Sub-optimal communication, including interpersonal interaction between trialists and patients, may have been a major factor in decision-making [16], sometimes leading to invalid consent. It should be borne in mind that patients enter trials with an innocence and fear that may be difficult for ‘expert’ trialists to comprehend [16]. This and the perceived inequality of levels of status, knowledge and power in the doctor/patient relationship [51] may obstruct a close alliance [42] and the means to clarify trial procedures. Our data show that patients felt undermined and separated from the medical enterprise. We suggest that patients may wish for a mode of caring that goes beyond ‘empathic information giving’ [52]. This raises questions as how best to formulate this interaction. We suggest it would involve physicians facilitating a context in which patients have closer contact and a ‘relationship’ with health personnel that includes trust and respect [41,42,53,54], together with a recognition that ill people experience vulnerability, and a need to feel ‘attached’ and included [41,42,53,54]. We have observed that the most engaged patients professed the most personal interaction with trial and other staff, leading to an enhanced patient experience. These factors are pertinent in the context of all RCTs, where ethical considerations incorporate the need for obtaining valid, informed and unbiased consent [17] and where the connectedness we speak of may help to restore autonomy and enhance recruitment.

### Limitations of the qualitative study

Our data apply to patients who are largely men of white ethnicity who have been invited to consent to a complex intervention trial which gave rise to problems that may not be encountered in straightforward RCTs. Our findings rely on patients’ perceptions and not the ongoing reality of a trial. We did not systematically investigate factors such as training levels or work overload of research staff, all of which may have impacted on our findings [38]. Qualitative studies that examine the ways trialists interact with potential participants in ‘real time’ may produce valuable insights [11]. Such a study was instigated midway through SPARE [47].

Despite these caveats, patients’ perceptions are important. The literature vindicates our findings, making it possible to extrapolate them to other RCTs.

## Conclusions

This study highlights the difficulty of providing balanced and clear trial information within the UK health system, despite the best intentions of research staff at participating sites. We suggest that not only is training made available to potential trialists but that consideration might be given to possible alternatives to randomization where complex interventions are being tested. Involvement of multiple professionals can impact on patient support, including communication processes with patients who are considering participation in RCTs, leading us to question the ‘deficit’ model of patients’ understanding and behavior. It is suggested that health professionals consider facilitating a context in which patients feel fully included in the trial enterprise.

## Abbreviations

CT: Chemotherapy; EUA: Examination under anaesthetic; Gem-cis: Gemcitabine – cisplatin chemotherapy; GP: General Practitioners; ICR: CTSU – Institute of Cancer Research – Clinical Trials and Statistics Unit; IQR: Interquartile range; NCRN: National Cancer Research Network; NHS: National Health Service; NIHR: National Institute for Health Research; PS: Performance Status; RCT: Randomized Controlled Trial; SBP: Selective Bladder Preservation; SPARE: A randomised trial of Selective bladder Preservation Against Radical Excision (cystectomy) in muscle invasive T2/T3 transitional cell carcinoma of the bladder; TCC: Transitional Cell Carcinoma; TMG: Trial Management Group; TUR: Transurethral Resection.

## Competing interests

The authors have no competing interests to declare.

## Authors’ contributions

CM is the primary author of this paper, designed the study in collaboration with AB, RH and EH, and was primarily responsible for data collection and analysis. RL and EJ assisted with data collection and analysis and contributed to the manuscript with AB, RH and EH. All authors read and approved the final manuscript.

## Authors’ information

CM is the Chief Investigator of the patient interviews study. RL is the Trial Manager for SPARE and EH is the Trial Statistician and is responsible for oversight of the management of the trial at ICR-CTSU. EJ is the SPARE Quality of Life Study coordinator. AB is the Clinical Co-ordinator and RH is the Chief Investigator of the SPARE trial.
